# Oligoether/Zwitterion Diblock Copolymers: Synthesis and Application as Cathode-Coating Material for Li Batteries

**DOI:** 10.3390/polym13050800

**Published:** 2021-03-05

**Authors:** Masahiro Yoshizawa-Fujita, Jun Ishii, Yuko Takeoka, Masahiro Rikukawa

**Affiliations:** Department of Materials and Life Sciences, Sophia University, 7-1 Kioi-cho, Chiyoda-ku, Tokyo 102-8554, Japan; jun-ishii@nisshinbo.co.jp (J.I.); y-tabuch@sophia.ac.jp (Y.T.); m-rikuka@sophia.ac.jp (M.R.)

**Keywords:** solid polymer electrolytes, zwitterions, RAFT polymerization, cathode, Li batteries

## Abstract

Poly (ethylene oxide) (PEO) has been investigated as an ion-conductive matrix for several decades due to its excellent properties. However, further improvements are needed to enable a PEO-based ion-conductive matrix for practical applications. In order to develop novel solid polymer electrolytes based on zwitterions, we synthesized diblock copolymers (PPEGMA-*b*-SPBs) with oligoether and zwitterionic side-chains and evaluated their physico-chemical properties. PPEGMA-*b*-SPBs with various unit ratios were synthesized by RAFT polymerization. PPEGMA-*b*-SPBs with/without LiTFSA exhibited two distinct glass transition temperatures regardless of the unit ratio of PEGMA and SPB. AFM observations clearly revealed phase-separated structures. The ionic conductivity of PPEGMA-*b*-SPBs increased even at a high salt concentrations such as [EO]:[Li] = 6:1 and was over 10^−5^ S cm^−1^ at 25 °C. This tendency is unusual in a PEO matrix. The oxidation stability of PPEGMA-*b*-SPBs was about 5.0 V vs. Li/Li^+^, which is a higher value than that of PEO. The improvement of the electrochemical properties is attributed to the introduction of the SPB block into the block copolymers. PPEGMA-*b*-SPBs were evaluated as cathode-coating materials for Li batteries. The discharge capacity and coulombic efficiency of the cells employing the cathode (LiNi_1/3_Mn_1/3_Co_1/3_O_2_ (NMC)) coated with the block copolymers were much higher than those of the cell employing the pristine cathode at the 50th cycle in the cut-off voltage range of 3.0–4.6 V.

## 1. Introduction

Poly (ethylene oxide) (PEO) has been investigated as an ion-conductive matrix for several decades due to its excellent properties, such as high polarity to dissociate Li-salts and flexibility to transport Li-ions along the polymer chain [1,2]. The ionic conductivity of PEO-based electrolytes is about 10^−4^ S cm^−1^ at room temperature. PEO can be utilized as a solid electrolyte for rechargeable batteries, e.g., Li-ion batteries. However, further improvements are needed to enable a PEO-based ion-conductive matrix for practical applications. Although an abundance of PEO derivatives have been reported, it is difficult to improve the ionic conductivity of PEO-based electrolytes. This is because the glass transition temperature (*T*_g_) increases and the ionic conductivity decreases as the concentration of Li-salt increases. In other words, there is a trade-off relationship between Li-ion concentration and Li-ion mobility. On the other hand, polycarbonate-based electrolytes have been reported as alternative solid polymer electrolytes [3,4,5]. Their ionic conductivity is about 10^−7^ to 10^−3^ S cm^−1^ at 60 °C, and the lithium transport number (*t*_Li+_) is typically in the range 0.4 to 0.8. Polycarbonate-based electrolytes are fascinating materials as an alternative to PEO, but the oxidation potential is around 3 V [4], which is lower than that of PEO-based electrolytes.

Additives are being actively studied in order to easily improve various properties of PEO. The ionic conductivity and *t*_Li+_ of PEO-based electrolytes and resistance at the electrolyte/electrode interface are improved by adding plasticizers [6,7] and inorganic fillers [8,9] to PEO-based electrolytes. On the other hand, zwitterions [10,11,12], which have both a cation and anion in the same molecule, have been proposed as new additives. The ionic conductivity and diffusion coefficient of Li-ion in polymer gel electrolytes are improved by the addition of zwitterions [13,14]. Furthermore, such improvements are also observed when zwitterions are added to ionic liquids, and the coulombic efficiency based on the electrochemical redox reaction of Li is improved [15].

The melting points of typical zwitterions are above 100 °C, hence zwitterions are solid at room temperature [11]. Recently, we succeeded in synthesizing zwitterions that are liquid at room temperature [16]. Furthermore, we found that liquid zwitterions improve the electrochemical (oxidation) stability of oligoether compounds [17,18]. Generally, the oxidation potential of PEO-based electrolytes is about 4.0 V vs. Li/Li^+^ [19,20], and cathode active materials such as LiCoO_2_ (LCO) and LiNi_1/3_Mn_1/3_Co_1/3_O_2_ (NMC) cannot be applied as high potential electrodes in Li-ion batteries with PEO-based electrolytes. Interestingly, when a small amount of zwitterion was added to oligoethers, the oxidation potential of oligoethers was improved to about 5 V. The combination of zwitterion and oligoether is interesting as an electrolyte material. However, oligoether electrolytes are liquids and have the same problems such as liquid leakage and volatility as organic electrolyte solutions. By developing polymer electrolyte membranes, not only can the above problems be solved, but also separator-free batteries can be fabricated. These allow us to develop safe and flexible devices. In order to develop novel solid polymer electrolytes based on zwitterions and oligoethers, we synthesized random copolymers with zwitterionic and oligoether side-chains [21]. Their lithium bis(trifluoromethanesulfonyl)amide (LiTFSA) composites exhibited an ionic conductivity of 10^−4^ S cm^−1^ at 80 °C. Contrary to conventional PEO-based electrolytes, their ionic conductivity was maintained even at high LiTFSA concentration because the high dipole moment of zwitterions leads to a dissociation of LiTFSA through electrostatic interaction [22,23].

There is a great interest in the use of microphase-separated block copolymers as solid electrolytes in rechargeable batteries [24]. Poly(styrene-*b*-oligoether)s have been proposed to satisfy both high ionic conductivity and excellent mechanical properties [25,26]. Poly(styrene-*b*-zwitterion)s have been reported as solid polymer electrolytes [27]. To the best of our knowledge, solid polymer electrolytes based on the block copolymers composed of zwitterionic and oligoether side-chains have not been reported. In this report, diblock copolymers with zwitterionic and oligoether side-chains are synthesized by reversible addition-fragmentation chain transfer (RAFT) polymerization. The thermal and electrochemical properties of the diblock copolymers are evaluated. The influence of unit ratio and Li-salt concentration on physico-chemical properties are investigated. Furthermore, Li batteries are fabricated to evaluate the diblock copolymers as cathode-coating materials because the oxidation stability of the diblock copolymers is improved to about 5 V, as with the oligoether electrolytes containing zwitterions (vide supra).

## 2. Materials and Methods

### 2.1. Materials

1,4-Dioxane (99.5%), hexane (96.0%), ethyl acetate (99.5%), and acetone (99.5%) were purchased from FUJIFILM Wako Pure Chemical Corp. (Chuo-ku, Osaka, Japan). The 1,1,1,3,3,3-Hexafluoro-2-propanol (HFIP) (99.0%) was purchased from Tokyo Chemical Industry Co., Ltd. (Chuo-ku, Tokyo, Japan). Poly (ethylene glycol) methyl ether methacrylate (PEGMA) (Sigma-Aldrich (St. Louis, MO, USA), 100 ppm MEHQ as inhibitor, 200 ppm BHT as inhibitor) was purified by alumina column (Merck KGaA, Ltd. (Darmstadt, Germany)) in order to remove inhibitor before use. [2-(Methacryloyloxy]ethyl)dimethyl-(3-sulfopropyl)ammonium hydroxide (SPB) (Sigma-Aldrich (St. Louis, MO, USA), 95%) was recrystallized in the mixed solvent (acetone:methanol = 10:1) before use. 4-Cyano-4-(phenylcarbonothioylthio)pentanoic acid (CTPA) (Sigma-Aldrich (St. Louis, MO, USA), 97.0%) was recrystallized in the mixed solvent (hexane:ethyl acetate = 2:3) before use. 2,2′-Azobis(isobutyronitrile) (AIBN) (FUJIFILM Wako Pure Chemical Corp. (Chuo-ku, Osaka, Japan)) and 4,4′-azobis(4-cyanovaleric acid) (ACVA) (FUJIFILM Wako Pure ChemicalCorp. (Chuo-ku, Osaka, Japan)) were recrystallized from methanol before use. *N*-Methyl-*N*-propylpyrrolidinium bis(fluorosulfonyl) amide ([P13][FSA]) was synthesized according to the procedure published in the literature [28]. Lithium bis(trifluoromethylsulfonyl)amide (LiTFSA) (Kanto Chemical Co., Inc. (Chuo-ku, Tokyo, Japan), 99.7%) and lithium bis(fluorosulfonyl) amide (LiFSA) (Kishida Chemical Co., Ltd. (Chuo-ku, Osaka, Japan), 99.0%) were dried at 100 °C in vacuo before use. Lithium foils (thickness: 0.4 mm, diameter: 16 mm) were purchased from Honjo Metal Co., Ltd. (Osaka, Japan). Poly(vinylidene difluoride) (PVDF) (#1120) was purchased from Kureha Battery Materials Japan Co., Ltd. (Chuo-ku, Tokyo, Japan). Acetylene black (AB) was purchased from Denka Ltd. (Chuo-ku, Tokyo, Japan). LiNi_1/3_Mn_1/3_Co_1/3_O_2_ (NMC) (average particle diameter: 0.81 µm) was purchased from Kusaka Rare Metal Products Co., Ltd. (Minato-ku, Tokyo, Japan).

### 2.2. Synthesis of PPEGMA

PEGMA (10.0 g, 2.00 × 10^−2^ mol), CTPA (39.2 mg, 1.40 × 10^−4^ mol), and AIBN (4.9 mg, 3.0 × 10^−5^ mol) were dissolved in 1,4-dioxane (30 mL) and placed in dried polymerization tubes. The polymerization mixtures were degassed with five pump thaw cycles, and then the tubes were sealed under nitrogen. The polymerization tubes were placed into a shaker at 70 °C. After 15 h, the reaction systems were cooled down in an ice bath. The polymer was purified by dialysis against pure water (18.1 MΩ cm^−1^) and recovered by freeze drying, yielding polymers (PPEGMA) as pink oils (yield: 91%). *M*_n_ (DMF, PEO/PEG standards) = 33,000 g mol^−1^, *M*_w_ (DMF, PEO/PEG standards) = 35,300 g mol^−1^, *M*_w_/*M*_n_ = 1.07. The polymerization degree (*n*) of PPEGMA*_n_* was determined to be 68. ^1^H NMR spectroscopy (D_2_O): 4.47–4.14 (3.98H, s), 4.02–3.53 (33.08H, m), 3.38–3.30 (3.04H, s), 2.34–1.71 (2.00H, m), 1.43–0.83 (12.96H, d). Analytical calculations for C_1496_H_2856_O_750_N_1_S_2_ (%): C, 54.77; H, 8.74; N, 0.04; S, 0.19; Found (%): C, 54.99; H, 9.04; N, 0.03; S, 0.04.

### 2.3. Synthesis of PPEGMA-b-SPB

The chain extension of PPEGMA_68_ to PPEGMA_68_-*b*-SPB*_m_* was carried out in a similar manner as RAFT polymerization of PPEGMA_68_. These copolymers were referred to as PPEGMA_68_-*b*-SPB*_m_*, where 68 and *m* represent the number of PEGMA and SPB units in the copolymers, respectively. SPB, PPEGMA_68_ (2.00 g, 6.06 × 10^−5^ mol) and ACVA (1.4 mg, 5.0 × 10^−6^ mol) were dissolved in pure water (18.1 MΩ cm^−1^) 25 mL and placed in dried polymerization tubes. The polymerization mixture was degassed with five pump thaw cycles, and then the tubes were sealed under nitrogen. The polymerization tubes were placed into a shaker at 70 °C. After 15 h, the reaction systems were cooled down in an ice bath. The polymer was purified by dialysis against pure water (18.1 MΩ cm^−1^) and recovered by freeze drying, yielding polymers as pink solids (general yields: 70–80%).

^1^H NMR spectroscopy (D_2_O): 4.62–4.28 (0.51H, s), 4.23–4.06 (0.52H, s), 3.89–3.61 (12.01H, m), 3.60–3.50 (1.15H, m), 3.49–3.29 (1.03H, s), 3.28–3.09 (3.02H, s), 3.01–2.87 (1.00H, s), 2.36–2.10 (1.00H, s), 2.09–1.68 (1.43H, m), 3.49–3.29 (2.22H, m). *M*_n_ (^1^H NMR, D_2_O) = 67,000 g mol^−1^. Analytical calculations for C_1496_H_2856_O_750_N_1_S_2_ (%): C, 50.99; H, 8.15; N, 2.55; S, 5.90; Found (%): C, 48.41; H, 8.28; N, 2.42; S, 5.76.

### 2.4. Preparation of Neat and Li Composite Films

In an Ar filled globe box, given amounts of PPEGMA_68_-*b*-SPB*_m_* and LiTFSA were dissolved in HFIP, and these mixtures were stirred at 25 °C for 24 h. Then, these solutions were cast on a PTFE plate and were dried at 25 °C for 24 h and 60 °C for 48 h under a nitrogen atmosphere. These films were dried in vacuo at 60 °C for 48 h before use. The LiTFSA concentration was varied from [EO]/[Li] = 6:1 to 24:1.

### 2.5. Methods

^1^H NMR measurements were carried out on a Bruker Avance III HD Nanobay 400 NMR spectrometer to confirm the chemical structures of products. Elemental analyses were carried out with a GC elemental analyzer (PE 2400-II, PerkinElmer, Inc. (Waltham, MA, USA)). The molecular weight and molar mass dispersity of PPEGMA_68_ were determined with gel permeation chromatography (GPC, PEO/PEG standard, Tosoh Corp. (Yamaguchi, Japan)) using DMF solution with 0.1 wt% LiCl as the eluent (1.0 mL min^−1^) at 40 °C.

The thermal properties were measured by thermogravimetric analysis (TGA; TG-DTA7200, Hitachi High-Tech Corp. (Minato-ku, Tokyo, Japan)). The samples were heated from 25 °C to 500 °C at a scan rate of 10 °C min^−1^ under a nitrogen atmosphere. The thermal behavior of the samples was measured with differential scanning calorimetry (DSC) (DSC7020, Hitachi High-Technologies Corp.) between −150 and 150 °C at a heating/cooling rate of 10 °C min^−1^.

The morphology of the film surface was measured by atomic force microscopy (AFM; SPM-9600, Shimadzu Corp. (Kyoto, Japan)). The AFM images were generated in the tapping mode with an amplitude ratio within 0.90–1.00 to avoid monolayer damage. The AFM cantilevers (NCHR-20, Nanoworld Co., Ltd. (Switzerland)) had spring constants in the range of 42 N m^−1^, the scanning range was 1 µm × 1 µm and the scanning rate was 1.0 Hz.

The ionic conductivity values of the samples were obtained by measuring the complex impedance between 100 mHz and 1 MHz (applied voltage: 10 mV) using an impedance analyzer (VSP-300, BioLogic (France)) over the temperature range of −40 to 100 °C. The temperature was controlled using a constant-temperature oven (SU-262, Espec Corp. Kita-ku, Osaka, Japan). A stainless steel cell (TYS-00DM01, Toyo System Co., Ltd. (Fukushima, Japan)) was used. Two platinum plates polished with 1.0, 0.3, and 0.05 μm Al_2_O_3_ powder were used as the electrode. The AC impedance method was used to analyze the ionic conductivity of the block copolymers at various temperatures. The ionic conductivity of the electrolytes was determined using

$$\sigma =\;\frac{d}{RS}$$

where *σ* represents ionic conductivity, *R* is the intercept at the real axis in the impedance Nyquist plot, *S* is the geometric area of the electrolyte-electrode interface, and *d* is the distance between the two electrodes.

Cyclic voltammetry measurements (CV; VSP-300, BioLogic (France)) were carried out in the potential range of −0.25 and 1.0 V at 60 °C at a scan rate of 1.0 mV s^−1^ with lithium foils as the reference and counter electrodes. A stainless steel cell (TYS-00DM01, Toyo System Co., Ltd. (Fukushima, Japan)) was used as the CV measurement cell. A nickel plate polished with 1.0, 0.3, and 0.05 μm Al_2_O_3_ powder was used as the working electrode. Linear sweep voltammetry (LSV) measurements (VSP-300, BioLogic (France)) were carried out in the potential range of 3.0 to 6.0 V at 60 °C at a scan rate of 1.0 mV s^−1^. Lithium foils were used as the reference and counter electrodes, while platinum plates were used as working electrodes in the potential ranges of 3.0 to 6.0 V.

NMC (85 wt%), AB (6 wt%), and PVDF (9 wt%) were mixed in *N*-methylpyrrolidone, then the composite solution was cast on the current collector, aluminum foil. The coated aluminum foils were dried at 80 °C for 1 h and were pressed at 6 MPa and 70 °C for 1 h. The coated aluminum foils were used as the pristine cathode. For application to cathode-coating material, PPEGMA_68_-*b*-SPB_121_ was applied onto the cathode. PPEGMA_68_-*b*-SPB_121_/HFIP solution (10 mg mL^−1^) was prepared. Spin coating (1H-D7, Mikasa (Minato-ku, Tokyo, Japan) was used for the fabrication of thin films to deposit a uniform coating of the block copolymer on the cathode surface and was performed at 3000 rpm for 30 s. The coated cathodes were dried at 60 °C for 48 h. Lithium foils were used as the anode. [C_3_pyr] [FSA]/LiFSA (0.32 mol kg^−1^) was used as the electrolyte for charge/discharge tests. Charge/discharge tests were carried out using a battery charge/discharge system (HJ-SD8, Hokuto Denko Corp. (Meguro-ku, Tokyo, Japan)). The cathode, anode, and separator (GA-55, Advantec (Chiyoda-ku, Tokyo, Japan)) soaked with the electrolyte were assembled in a stainless steel cell (TYS-00DM01, Toyo System Co., Ltd.) in an Ar-filled glove box (H_2_O < 1 ppm, O_2_ < 1 ppm) to minimize moisture contamination.

## 3. Results and Discussion

### 3.1. Synthesis of Oligoetehr/Zwitterion Diblock Copolymers

**Figure 1:**
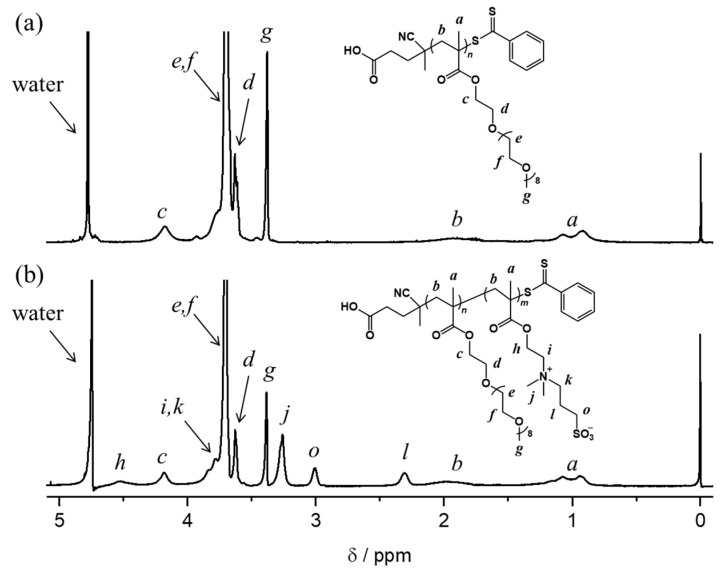
^1^H NMR spectra of (**a**) PPEGMA_68_ and (**b**) PPEGMA_68_-b-SPB_121_ in D_2_O.

A homopolymer of PEGMA, PPEGMA was synthesized by RAFT polymerization in 1,4-dioxane. The number-average molar mass (M_n_) and mass-average molar mass (*M*_w_) were determined by GPC measurements to be 33,000 and 35,300 g mol^−1^, respectively. The molar mass dispersity (Ð_M_) was calculated from the molecular weight values to be 1.07, indicating that the molecular weight of PPEGMA is well-controlled by RAFT polymerization. The polymerization degree (n) of PPEGMA_n_ was also calculated from the M_n_ value to be 68. Figure 1a shows the ^1^H NMR spectrum of PPEGMA_68_ in D_2_O. The chemical shifts of the methacrylate group were observed at about 1.0 and 2.0 ppm. The chemical shifts, which are assigned to the oligoether side-chain of PEGMA, were observed in the range of 3.0 to 4.0 ppm. These results confirm the successful synthesis of PPEGMA.

We synthesized four kinds of diblock copolymers, PPEGMA_68_-*b*-SPB_m_, with various SPB unit numbers as summarized in Table 1. The number-averaged molar mass (M_n_) of PPEGMA_68_-*b*-SPB_m_ was determined by ^1^H NMR measurements to be in the range of 37,800 and 67,000 g mol^−1^. Figure 1b shows the ^1^H NMR spectrum of PPEGMA_68_-*b*-SPB_121_ in D_2_O. The chemical shifts of the zwitterionic side-chain were observed in the range of 2.2 to 4.0 ppm. From these results, the block copolymers composed of PEGMA and SPB were successfully obtained by RAFT polymerization. The SPB unit numbers (m) were calculated from the M_n_ values of diblock copolymers and were in the range of 17 and 121. The ratio of m and n (m/n) was determined to be from 0.25 to 1.8.

### 3.2. Thermal Properties

**Figure 2:**
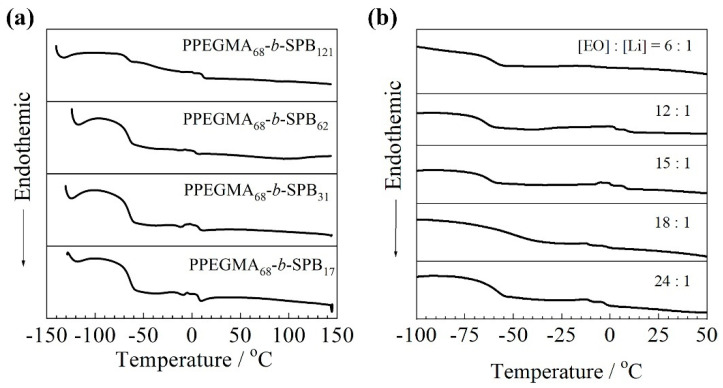
DSC traces of (**a**) PPEGMA_68_-*b*-SPB_m_ and (**b**) PPEGMA_68_-*b*-SPB_121_/LiTFSA composites (2nd heating).

DSC measurements were performed on the diblock copolymers in order to detemine the thermal properties. Figure 2 shows (a) DSC curves of neat diblock copolymers, PPEGMA_68_-*b*-SPB_m_, and (b) PPEGMA_68_-*b*-SPB_121_/LiTFSA composites at the 2nd heating scan. All the neat diblock copolymers exhibited two distinct glass transition temperature (T_g_) values at around −75 and 5 °C as shown in Figure 2a. The T_g_ values are also summarized in Table 1. The lower T_g_ (T_g,L_) is almost the same with the T_g_ (−74 °C) of PPEGMA [29]. Galin et al. reported that a random copolymer composed of butyl acrylate and zwitterionic monomer exhibited two distinct T_g_ values at −38 and 44 °C, which corresponded to buryl acrylate and zwitterion, respectively [23]. The lower T_g_ and higher T_g_ should be based on PEGMA and SPB units, respectively. Both T_g_,_L_ and T_g_,_H_ exhibited almost the same values regardless of the unit ratio, m/n. These results indicate that PPEGMA and SPB are incompatible components and tend to spontaneously form phase-separated structures. AFM observation was performed to investigate phase-separated structures for PPEGMA_68_-*b*-SPB_m_. Microphase separation was observed for all the block coplymers (data not shown). The SPB block formed an aggregation in the matrix due to strong interaction between zwitterionic side-chains.

PPEGMA_68_-*b*-SPB_121_/LiTFSA composites also exhibited two distinct *T*_g_ values at around −70 and 5 °C except for [EO]:[Li] = 6:1 as shown in Figure 2b. The *T*_g,L_ values of PPEGMA_68_-b-SPB_121_/LiTFSA with [EO]:[Li] = 24:1, 18:1, 15:1, 12:1, and 6:1 were −66, −65, −67, −69, and −67 °C, respectively. The *T*_g_,_L_ of PPEGMA_68_-*b*-SPB_121_/LiTFSA was about 10 °C higher than that of the neat block copolymer. This increase is assumed to be mainly based on the interaction between the ether oxygen of PEGMA units and Li^+^. Interestingly, the *T*_g,L_ exhibited a constant value in the Li-salt concentration range of [EO]:[Li] = 24:1 and 6:1. The *T*_g_ of PEO-based electrolytes generally increases with increasing salt concentration due to the interaction (vide supra). On the other hand, the *T*_g,H_ values of PPEGMA_68_-*b*-SPB_121_/LiTFSA with [EO]:[Li] = 24:1, 18:1, 15:1, and 12:1 were −3, −2, 7, and 8 °C, respectively. The *T*_g_,_H_ of PPEGMA_68_-*b*-SPB_121_/LiTFSA was lower than that of the neat block copolymer. A low molecular weight zwitterion, 4-(1-ethyl-1H-imidazol-3-ium-3-yl)butane-1-sulfonate, exhibits a *T*_g_ at 18 °C. Interestingly, the equimolar mixture of the zwitterion and LiTFSA forms a liquid at room temperature and exhibits a *T*_g_ at −37 °C [11]. The aggregation state of the SPB block will be plasticized by the addition of LiTFSA.

### 3.3. Surface Morphology

**Figure 3:**
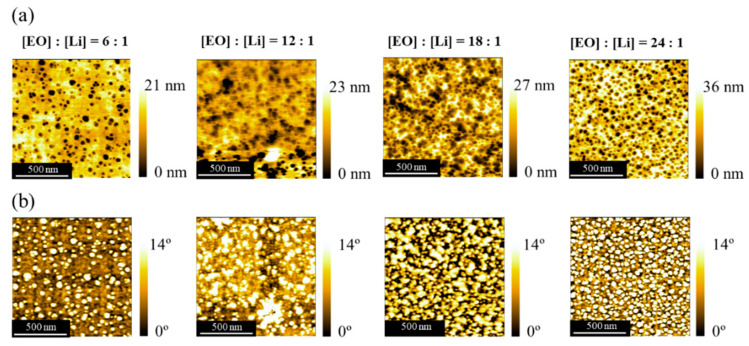
AFM (**a**) height and (**b**) phase images of PPEGMA_68_-*b*-SPB_121_/LiTFSA composites.

The surface morphology was investigated by AFM measurements for PPEGMA_68_-*b*-SPB_121_ with various LiTFSA concentrations ([EO]:[Li] = 6:1, 12:1, 18:1, and 24:1). Figure 3 shows AFM (a) height and (b) phase images of PPEGMA_68_-*b*-SPB_121_/LiTFSA composites. Phase-separated structures were observed for both height and phase images of all the composites. In the AFM height images, round dark domains are the SPB block according to the volume ratio of SPB block and PEGMA block. The number of the round dark domains decreased with increasing LiTFSA concentration. In addition, the surface roughness decreased with increasing LiTFSA concentration. The AFM phase images also showed the same phase-separated structure as the height images. The aggregation of SPB block was observed as round bright domains in the phase images. The number of round bright domains decreased with increasing LiTFSA concentration. These results suggest that the SPB block was plasticized by the addition of LiTFSA, and the phase-separated structure was relaxed due to the mixture formation of SPB and PEGMA blocks. This tendency is in good accordance with the results of thermal properties.

### 3.4. Ionic Conductivity

**Figure 4:**
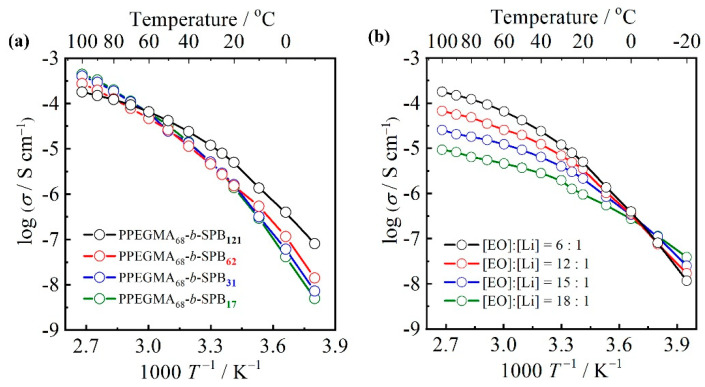
Arrhenius plots of ionic conductivities for (**a**) PPEGMA_68_-*b*-SPB_m_/LiTFSA composites ([EO]:[Li] = 6:1) and (**b**) PPEGMA_68_-*b*-SPB_121_/LiTFSA composites.

The effect of the unit number of SPB on the ionic conductivity was investigated for PPEGMA_68_-*b*-SPB_m_ (m = 17, 31, 62, and 121). The Li-salt concentration was fixed at [EO]:[Li] = 6:1. Figure 4a shows the Arrhenius plots of ionic conductivities for PPEGMA_68_-*b*-SPB_m_/LiTFSA composites. The temperature dependence of ionic conductivities exhibited upper convex curves for all PPEGMA_68_-*b*-SPB_m_/LiTFSA composites, suggesting that the ion motion in the block copolymers is mainly coupled with the segmental motion of PPEGMA matrix, which is the typical ion conduction mechanism in a PEO matrix [1,2]. The ionic conductivities of PPEGMA_68_-*b*-SPB_17_ and PPEGMA_68_-*b*-SPB_31_ were almost the same within the temperature range measured in this study. The two block copolymers exhibited the highest ionic conductivity at higher temperatures above 60 °C in this study. On the other hand, at lower temperatures below 60 °C, the ionic conductivity of block copolymers increased with increasing the unit number of SPB. The ionic conductivity is governed by the number of carrier ions and carrier ion mobility. This tendency would result in the enhancement of Li-salt dissociation by SPB units, which has high polarity [22,23]. As a result, a large number of carrier ions is provided into the block copolymer matrix. Thus, PPEGMA_68_-*b*-SPB_121_, which has a longer SPB block, exhibited higher ionic conductivity than those of the other block copolymers especially at lower temperature range below 60 °C. In other words, PPEGMA_68_-*b*-SPB_121_ exhibited a gentle slope (lower activation energy) for the Arrhenius plot of ionic conductivity, suggesting that the polarity of the zwitterion structure prevents the aggregation of dissociated ions by the enhancement of Li-salt dissociation.

The effect of the Li-salt concentration on the ionic conductivity was investigated for PPEGMA_68_-*b*-SPB_121_/LiTFSA composite, which exhibited the highest ionic conductivity of 10^−5^ S cm^−1^ at 25 °C among the four block copolymers. Figure 4b shows the Arrhenius plots of ionic conductivities for PPEGMA_68_-*b*-SPB_121_/LiTFSA composites. The Li-salt concentration was changed in the range of [EO]:[Li] = 6:1 and 18:1. The ionic conductivity of PPEGMA_68_-*b*-SPB_121_/LiTFSA composites increased with increasing Li-salt concentration from [EO]:[Li] = 18:1 to 6:1. In general, PEO-based electrolytes show the highest ionic conductivity at around [EO]:[Li] = 20:1 [3,30] because the introduction of a large amount of Li-salt into the PEO matrix induces an increase in T_g_, namely, the decrease in segmental motion of PEO matrix, which results in the decrease in ionic conductivity. PPEGMA_68_-*b*-SPB_121_/LiTFSA composites exhibited an unusual tendency for Li-salt concentration dependence on ionic conductivity. This is attributed to the high polarity of the zwitterion structure in the SPB units enhancing the Li-salt dissociation (vide supra).

### 3.5. Electrochemical Properties

**Figure 5:**
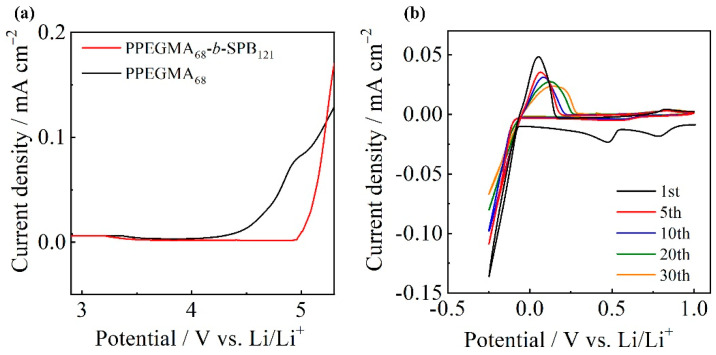
(**a**) Linear sweep voltammograms of PPEGMA_68_/LiTFSA and PPEGMA_68_-*b*-SPB_121_/LiTFSA at 60 °C (scan rate: 1 mV s^−1^; working electrode: Pt; counter and reference electrodes: Li), and (**b**) cyclic voltammograms of PPEGMA_68_-*b*-SPB_121_/LiTFSA at 60 °C (scan rate: 10 mV s^−1^; working electrode: Ni; counter and reference electrode: Li).

The electrochemical stability of electrolyte materials is an important factor in determining the battery performance. To determine the effect of the SPB block on the electrochemical stability of the diblock copolymers, linear sweep voltammetry (LSV) measurements were carried out at 60 °C. Figure 5a shows the LSV results of PPEGMA_68_/LiTFSA and PPEGMA_68_-*b*-SPB_121_/LiTFSA (anodic scan from 2.9 to 5.6 V). The oxidation potential of PPEGMA was approximately 4.2 V vs. Li/Li^+^, which is identical to the value reported previously for PEO-based electrolytes [31]. On the other hand, the oxidation potential of PPEGMA_68_-*b*-SPB_121_ was approximately 5.0 V vs. Li/Li^+^. The oxidation limit was improved from 4.2 to 5.0 V by the introduction of SPB block in the copolymer. We have already reported that the oxidation limit of oligoether electrolytes is improved from around 4.5 to over 5.0 V vs. Li/Li^+^ by the addition of zwitterions [17,18]. The effect of zwitterion on the interfacial structures of Pt/tetraglyme (G4)-LiTFSA systems was investigated using in-situ infrared-visible sum frequency generation (IV-SFG) spectroscopy [32]. The SFG spectra confirmed that the Pt surface is covered by the Li^+^/zwitterion complex cations, which are more stable to oxidation than free G4 molecules. The enhancement of electrochemical stability of PPEGMA_68_-b-SPB_121_ is in good accordance with the results of G4 system, suggesting that the formation of Li^+^/SPB complex results in full coverage of the SPB block at the Pt electrode surface which accounts for the enhanced oxidation stability of the block copolymers.

Figure 5b shows the CV results of PPEGMA_68_-*b*-SPB_121_/LiTFSA composites on Ni electrodes at 60 °C. Continuous anodic current and two reduction peaks at 0.5 and 0.8 V vs. Li/Li^+^ were observed only at the 1st anodic scan. These anodic current and reduction peaks will be associated with the reactions of oxides and water remaining on electrode suface [33]. The voltammograms of the composite electrolyte showed a reduction peak at −0.2 V vs. Li/Li^+^ and an oxidation peak at 0.1 V vs. Li/Li^+^ for Li at the 1st cycle. These reduction and oxidation peaks were observed over 30 cycles. However, the current density of the peaks decreased with increasing the number of cycles. In addition, the maximum current density of the oxidation peak shifted to a higher potential value and was broadened with an increasing number of cycles. These observations has been attributed to the formation of a suitable solid electrolyte interphase (SEI) film on the electrode surface [34]. The stable stripping/plating reactions were observed over 30 cycles, indicating that the block copolymers composed of PEGMA and SPB function as a Li-ion conductor.

### 3.6. Charge/Discharge Properties

**Figure 6:**
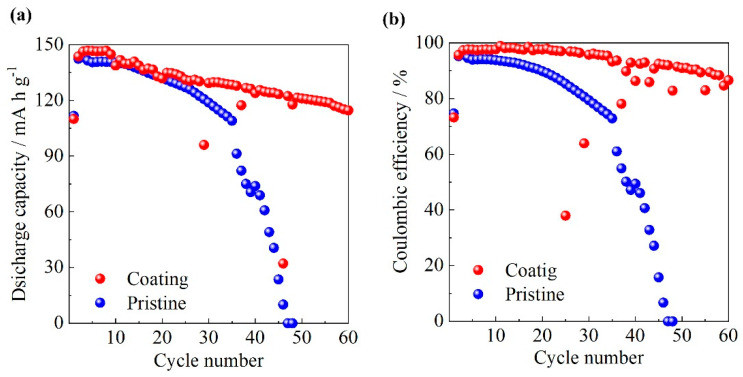
(**a**) Discharge capacities and (**b**) coulombic efficiencies of Li/NMC cells containing [C_3_mpyr] [FSA] over 60 cycles in the cut-off voltage range of 3.0–4.6 V at 60 °C (Charge/discharge rate: 0.5 C).

Figure 6 shows (a) the discharge capacities and (b) coulombic efficiencies of the Li/electrolyte/NMC cells (electrolyte: [C_3_mpyr] [FSA]/LiFSA (0.32 mol kg^−1^)) over 60 cycles in the cut-off voltage range of 3.0–4.6 V. The cathode surface was coated by PPEGMA_68_-*b*-SPB_121_. Pristine cathode was also used for comparison. The discharge capacity of the cell employing the pristine cathode was 140 mAh g^−1^ during the first 10 cycles and gradually decreased with an increasing number of cycles. Finally, the cell exhibited no discharge capacity at the 50th cycle. The coulombic efficiency of the cell employing the pristine cathode exhibited the same tendency with the discharge capacity and was maintained at 95% during the first 10 cycles. The coulombic efficiency decreased with increasing cycle number and reached 0 % at the 50th cycle. On the other hand, the discharge capacity of the cell employing the cathode coated with PPEGMA_68_-*b*-SPB_121_ exhibited a constant value of 145 mAh g^−1^ during the first 10 cycles and gradually decreased with increasing cycle number, from 145 mAh g^−1^ to 120 mAh g^−1^ at the 60th cycle. The coulombic efficiency of the cell employing the cathode coated with the block copolymer exceeded 90% over 60 cycles. The discharge capacity and coulombic efficiency of the cell employing the coated cathode were much higher than those of the cell employing the pristine cathode at the 50th cycle. The coating of PPEGMA_68_-*b*-SPB_121_ on the cathode surface was effective to lower the rate of cell capacity and coulombic efficiency fading in the cells at high cut-off voltage values. The effect of zwitterions on charge/discharge properties of Li/electrolyte/LiCoO_2_ cells with oligoether and ionic liquid electrolytes has been reported in the same cut-off voltage range of 3.0–4.6 V [17,28,35]. Although the discharge capacities and coulombic efficiencies of the cells employing the electrolytes without zwitterions decreased with cycle number, the addition of zwitterions into the electrolytes was effective to stablilize the discharge capacities and coulombic efficiencies during 50 cycles. This should be based on the improvement of oxidation stability of electrolyte materials by the addition of zwitterions. In the cells employing the cathode coated by the block copolymers, the SPB block on the electrode surface also improves the oxidation stability of electrolyte materials at higher cut-off voltages. In general, the discharge capacity of cells employing cathode active materials such as NMC and LCO increases with increasing cut-off voltage value. This type of material, which prevents the decomposition of electrolyte materials at higher voltages, is intended for the development of deep discharge batteries and will open a new methodology to improve the charge/discharge properties of Li batteries.

## 4. Conclusions

The block copolymers, PPEGMA_68_-*b*-SPB*_m_* (*m* = 17, 31, 62, and 121), with well-controlled molecular weights were synthesized by RAFT polymerization. The *M*_n_ of PPEGMA_68_-*b*-SPB*_m_* was determined by ^1^H NMR measurements to be in the range of 37,800 and 67,000 g mol^−1^. All the neat diblock copolymers exhibited two distinct *T*_g_ values at around −75 and 5 °C, which are associated with PEGMA and SPB units, respectively. PPEGMA_68_-*b*-SPB*_m_*/LiTFSA composites also exhibited two distinct *T*_g_s except for [EO]:[Li] = 6:1. PEGMA and SPB are incompatible components and tend to spontaneously form phase-separated structures. Microphase separation was observed for all the block copolymers and their LiTFSA composites in AFM observation. PPEGMA_68_-*b*-SPB_121_/LiTFSA composites exhibited higher ionic conductivities than those of the block copolymers with lower SPB contents. The ionic conductivity of PPEGMA_68_-*b*-SPB_121_/LiTFSA composites increased with increasing LiTFSA concentration. These results would result in the enhancement of Li-salt dissociation by SPB units, which has high polarity. The oxidation potential of PPEGMA_68_-*b*-SPB_121_ was approximately 5.0 V vs. Li/Li^+^. The oxidation limit was improved by the introduction of the SPB block in the PEO-based copolymers. The stable stripping/plating reactions of Li were observed over 30 cycles in CV measurements. The block copolymers were composed of PEGMA, and SPB functions as a Li-ion conductor. The Li/NMC cells were fabricated by using an NMC surface coated with PPEGMA_68_-*b*-SPB_121_. The discharge capacity and coulombic efficiency of the cells exhibited higher values at the 60th cycle as compared with those of the cells with pristine NMC in the cut-off voltage range of 3.0–4.6 V. The SPB block on the electrode surface improves the oxidation stability of electrolyte materials at higher cut-off voltages. Zwitterions function effectively as cathode-coating materials in Li batteries. This will be a new methodology to improve charge/discharge properties of not only Li batteries but also other rechargeable batteries.

## Figures and Tables

**Table 1:** Molecular Weights (*M*_n_) and Glass Transition Temperatures (*T*_g_) of PPEGMA_68_-*b*-SPB_m_.

Sample	*M*_n_*^a^*/g mol^−1^	*m ^b^*	*m/n ^c^*	*T*_g,L_*^d^*/°C	*T*_g,H_*^e^*/°C
PPEGMA_68_-*b*-SPB_121_	67,000	121	1.8	−78	10
PPEGMA_68_-*b*-SPB_62_	50,500	62	0.91	−76	2
PPEGMA_68_-*b*-SPB_31_	42,000	31	0.46	−70	7
PPEGMA_68_-*b*-SPB_17_	37,800	17	0.25	−70	6

*^a^* Determined by ^1^H NMR, *^b^* SPB unit number, *^c^* Unit ratio of SPB and PEGMA, *^d^* Lower glass transition temperature, *^e^* Higher glass transition temperature.

## Data Availability

The data presented in this study are available on request from the corresponding author.
